# Researches into the Structure of the Nervous System

**Published:** 1844-07

**Authors:** 


### ART. XI.

1. *Untersuchungen über den Bau des Nerven-systems.* Von Dr. B. STILLING und Dr. J. WALLACH. *Zweites Heft, enthaltend Untersuchungen über die Textur und Function der Medulla Oblongata.* Von Dr. B. STILLING. Mit 7 Tafeln abbildungen.—*Erlangen,* 1843.
*Researches into the Structure of the Nervous System.* By Dr. B. STILLING and Dr. J. WALLACH. *The Second Part, containing Researches into the Structure and Function of the Medulla Oblongata.* Seven Plates.—*Erlangen,* 1843. 4to, pp. 72.

2. *Mikroskopiske Undersögelser af Nervensystemet.* Ved ADOLPH. HANNOVER, Lic. Med.—*Kjöbenhavn,* 1842.
*Microscopical Investigations of the Nervous System.* By Dr. ADOLPH. HANNOVER. With Plates.—*Copenhagen,* 1842. 4to, pp. 112.

3. *Untersuchungen über die Physiologie der Nervenfaser.* Von Dr. G. H. MEYER, Privat-docenten zu Tübingen, &c.—*Tübingen,* 1843.
*Researches into the Physiology of Nervous Fibrils.* By Dr. G. H. MEYER, &c. 8vo, pp. 316.

IN a former article (see our last Vol., p. 379) we reviewed some of the recent discoveries in the anatomy and physiology of the sympathetic system, and of the spinal cord. In the present, we propose to trace those facts respecting the structure and function of the medulla oblongata and encephalon, which the works before us may furnish.

I. DR. STILLING'S RESEARCHES. Dr. Stilling divides the medulla oblongata into four portions: the first extends from the origin of the second pair of cervical nerves to the roots of the hypoglossal nerve; the second is that portion circumscribed between the lowest twig of the hypoglossal nerve, and the lowest of the vagus; the third is that part between the last-mentioned point, and the origin of the glosso-pharyngeus; and the fourth from hence to the pons varolii. The general organization, and the special microscopic anatomy of each of these portions are detailed at length.

Dr. Stilling observes that the structure of the spinal cord, as described in our last Volume, is the same throughout upwards to the origin of the spinal accessory nerve; it is at this point that a change is first observable. The organization is also similar from the origin of the seventh pair of cervical nerves to that of the second; but from the insertion of the middle and inferior roots of this nerve, as far as the pons varolii, the organization of the cord gradually changes; so that these two points are the true limits of the medulla oblongata.

To state generally the results of Dr. Stilling's researches, we remark, that the longitudinal fibres of the white matter in the anterior, posterior, and lateral strands of the cords have an uninterrupted course from the cauda equina to the pons varolii, and beyond it; and so also the longitudinal fibres of the gray substance and of the gelatinous substance. How they terminate in the *cauda equina* is not known. The white fibres which form the lateral strands lie upon one another, and are distinct. The "spinal bodies" are situate among the fibrils of the anterior gray substance only along their whole course, and are collected in larger masses where the nerves of the extremities pass off. The longitudinal fibres of both kinds are also traversed by horizontal fibrils, which enter the posterior strands at the roots of the posterior nerves, cross the posterior longitudinal fibres of the white, gray, and gelatinous substance, and, after sending a part to the opposite lateral half behind and before the spinal canal, pass on in a direct horizontal direction through the spinal bodies, and crossing the white and gray longitudinal fibrils of the anterior columns emerge as the roots of the anterior nerves. The prolongation of the pia mater and the blood-vessels dip deep into the cord, and are accompanied by remarkably fine gray fibrils given off from the anterior gray matter, and are considered by Dr. Stilling to be nervi vasorum. In man, at several points where the gray and white matter of the lateral strands join, gray fibrils dip (but not deeply) into the white. These latter are principally seen at the enlargement of the cord, corresponding to the roots of the nerves of the arm. In the medulla oblongata there are additional elements :—1, new masses of gray substance, and new white longitudinal fibrils ; 2, a new species of semicircular fibrils, running in a longitudinal direction, make their appearance ; and 3, an important intermixture of white and gray fibrils unlike anything in the spinal cord. The gray masses are found on all the strands, and around the spinal canal ; in the anterior columns they form the corpora pyramidalia ; in the lateral, the corpora olivaria ; in the posterior, the corpora restiformia, the funiculi graciles, and funiculi cuneati ; and around the spinal canal, the nuclei (kerne) of the hypoglossus, vagus, accessorius, and glosso-pharyngeus. The new white fibrils are peculiar to the anterior strands. They commence near to the point where the medulla oblongata begins, namely, between the first cervical pair and the hypoglossus. These arising from the anterior gray commissure at the margin of the anterior sulcus, are applied to the white matter of the anterior column, and pass upwards to and through the pons varolii. The new semicircular and horizontal fibres spring from the nuclei below and behind the spinal canal, and traverse the posterior, lateral, and anterior columns. They form the fibræ transversæ and fibræ arciformes, described by German anatomists, as being in relation with the inferior surface of the corpora olivaria. Besides these changes in the medulla oblongata, its primary white and gray longitudinal fibres are more intermixed than the similar structures of the medulla spinalis, and this intermixture is so much increased by the introduction of the semicircular fibrils, that a most complete network is formed. The fibres of the hypoglossus, accessorius, vagus, and glosso-pharyngeus nerves pass inwards between these various kinds of fibrils as far as the gray matter round the spinal canal (the floor of the fourth ventricle) each to their corresponding nucleus, the hypoglossus to the anterior gray mass or nucleus, the accessorius, vagus, and glosso-pharyngeus to the posterior and lateral. The course of these nerves is analogous to that of the spinal nerves, and the relation of the hypoglossus to the other three is the same as that of the anterior roots of the spinal nerves to the posterior. The anterior sulcus is rendered shallower by the accession of the pyramidal fibres, and by the intimate intermixture of the white and gray fibrils, and at last disappears. There is no canal in the medulla oblongata the greater part of its length ; the continuation of the spinal canal reappears, however, as the commencement of the fourth ventricle. At the point where the medulla oblongata commences, the two halves cease to be symmetrical. The greater part of the white substance of the anterior column appears forced from right to left, and pressed into the right lateral half of the medulla oblongata. Higher up between the superior root of the first pair of cervical nerves and the lowest root of the hypoglossus, the symmetrical appearance diminishes, and less white matter is seen on the right side. Can this increase in size if the left half have any connexion with the heart's action? or is it not rather connected with the *right* muscular half of the trunk? The elementary fibrils of the medulla oblongata resemble those of the spinal cord; but the spinal bodies in the nucleus of the hypoglossus alone resemble those in the anterior gray matter of the medulla spinalis. Those in the nuclei of the vagus, accessorius, and glosso-pharyngeus, are however much smaller, less than one half the size; and can only be seen as fine points with the aid of a glass magnifying fifteen times, while the "spinal bodies" may be seen with the naked eye, when a very thin slice is held up to the light. The smaller differ in size, those in the nuclei of the vagus and accessorius being the smallest. They contain a nucleus like the larger, and show a greater number of longer or shorter spines.

*Microscopic anatomy of the respiratory nerves in the medulla oblongata.* Dr. Stilling traces the microscopic anatomy of the medulla oblongata and of the nerves proceeding from it according to the four sections we before described. He gives a general and detailed description of the structure of each section, noticing the origin of the nerves, and the changes in the arrangement of the elementary constituents. We shall describe the origin of the nerves only, leaving the other details for the special attention of those of our readers who take interest in histological researches.

*Nervus accessorius Willisii.* At the commencement of the medulla oblongata, the fibrils of the posterior nerves, after traversing the substantia gelatinosa, pass through the lateral strands, (which now contain gray fibres,) and form a complicated network with the longitudinal fibrils. From this network, bundles of fibrils, which appear silvery under the microscope, are seen to pass towards the posterior surface of the lateral strands at a little distance from the point of insertion of the posterior nerves. Sometimes there is only one thick bundle, sometimes two, three, or more thinner bundles; they are composed of fibrils resembling the other transverse fibrils that are the continuation of the posterior nerves, and they are unmistakably seen to be the prolongation of a part of the nervus accessorius willisii. These fibrils differ, however, from those of the posterior roots of the second and third pair of cervical nerves in this, that whereas the latter penetrate the substantia gelatinosa, the fibrils of the spinal accessory do not touch it, but are separated from it by bundles of white longitudinal fibrils. The other root of this nerve passes transversely inwards to the new gray matter already described near the canalis spinalis, and which is the point whence the fibrils of the nerves of this part diverge. This canal is not now in the centre or axis of the cord, but is approximated to the posterior surface, getting nearer and nearer as it ascends towards the brain, until it forms the commencement of the fourth ventricle.

*Origin of the hypoglossus.* Anteriorly to the canalis spinalis, and partly on both sides, there is a congeries of larger "spinal bodies," constituting a round, oval, or heart-shaped nucleus or ganglion. Bisected it forms two cylinders, situate anteriorly to the spinal canal, and the one to the right, the other to the left. From this cylindrical mass, the hypoglossal nerves of each side proceed. These nuclei approximate the posterior periphery of the medulla oblongata *pari passu* with the canalis spinalis, and appear on the floor of the fourth ventricle, one being on each side of the groove in the calamus scriptorius.

*The accessorius ganglion or nucleus,* is situate below and behind the spinal canal, and is most intimately connected with the preceding by fine fibrils; it is the analogue of the posterior commissure, as the hypoglossal nucleus is the analogue of the anterior. This ganglion increases also in size from below upwards; it is of a crescentic form, the centre of the crescent being filled with white matter, and has two pointed extremities, an anterior or inner, and a posterior or outer. From the inner extremity bundles of fibrils pass directly outwards with a slight inclination forward, and appear on the surface of the medulla as the superior root of the spinal accessory nerve. The outer extremity gives off fibrils, a few of which join the preceding, but the greater number form arcs, as they pass forwards and inwards, crossing with radical fibres of the accessorius, and intersecting those of the opposite side, and of the hypoglossus, and uniting in the middle line. The two accessorius' nuclei form the lateral boundaries or walls of the fourth ventricle.

The origin from the anterior gray matter of the fibrils composing the pyramid may be seen with the naked eye, if a longitudinal section be made through the portion of the medulla named. Dr. Stilling agrees with Rolando in stating, in opposition to a host of authorities, that there is no commissural union of the anterior pyramids, no white fibrils of the anterior column decussated from right to left, or *vice versâ.* Two nuclei (kerne) are described as existing within the pyramids, which Dr. Stilling names the greater and the less.

*Origin of the vagus, and its nucleus.* The fibrils of the vagus pass horizontally into the medulla, and enter the mass of gray substance which rests on the floor of the fourth ventricle, and is known as the alæ cinereæ; this is the nucleus or ganglion of the vagus. It is a direct continuation of the nucleus of the spinal accessory, which it closely resembles in form, in the mode in which the fibrils pass off, and in structure; only it is much finer and softer, presenting a peculiar, gelatinous, bluish, semi-transparent appearance. Its inner margin is in relation with the nucleus of the hypoglossus with which it is most closely connected, and it passes so insensibly into the nucleus of the accessorius, that the limits of the two are not perceptible.

*The glosso-pharyngeus, and its nucleus.* The roots of this nerve arise like those of the preceding, from a mass of gray matter situate, internally, and posteriorly, in the medulla oblongata. Both the nucleus and its radiating fibrils have the most intimate union with the preceding.

*Anatomy of the fourth ventricle.* According to Dr. Stilling, the floor of the fourth ventricle is made up principally by the nuclei just described. First, at the inferior extremity, embracing the point of the calamus scriptorius, there is a crescentic flat mass of gray substance, having a soft reddish appearance, from which, on each side, the funiculi graciles and funiculi cuneati diverge. This is the nucleus of the spinal accessory nerve. Adjoining to and just above this, on each side, there is a triangular mass of gray substance, whose points form the points of the calamus scriptorius, and whose bases project, and are directed upwards and outwards. This is the nucleus of the vagus. On each side, close to the middle line is situate the hypoglossal nucleus, its outer margin is applied to the nucleus of the vagus nerve; its inner forms the slit of the calamus; it is also triangular in shape, the point being downwards, and the base upwards. Close to the base of the triangular mass of gray matter which forms the nucleus of the vagus, is another triangular mass; this is the glosso-pharyngeal nucleus; superiorly and externally is the continuation of the funiculus gracilis, and funiculus cuneatus. It ceases at the point where the transverse fibres of the acoustic nerve rests on the floor of the ventricle.

There is a short notice of the anatomy of the medulla oblongata in the calf. The organization is in general similar to that in man. Dr. Stilling notices, however, the following points of difference. In the calf, the two sides are perfectly symmetrical; the nucleus of the larger pyramid is larger than in man, and a section presents two or three sigmoid convolutions; there are no new pyramid-fibres, and the pyramids themselves and the olivary bodies are less strikingly developed. The sulcus is less deep, and the white portions do not interlace as in man.

*Functions of the medulla oblongata and its nerves.* Dr. Stilling attributes two principal offices to the medulla oblongata, similar indeed to those of the spinal cord. 1. It excites and maintains the energy of the nerves derived from it. 2. It receives impressions from the sensitive nerves and conducts them, by means of the longitudinal and transverse fibrils in every direction, upwards to the brain, and downwards to the cord, thereby exciting those parts; or it distributes a stimulus and excites an activity within its own structure, and through this, in the nerves arising from it. In the spinal cord, the close relations of the substantia gelatinosa, and of the "spinal bodies" to the sensitive and motor nerves lead to the inference that the former is subservient to sensation, and the latter to motion. So in the medulla oblongata, we find the nucleus corresponding to the layers of "spinal bodies," and the nuclei of the accessorius, vagus, as glosso-pharyngeus correspond to the substantia gelatinosa; so that the latter have a sensitive, the former a motor function. But indeed, the accessorius is of a mixed character; while the inferior and middle twigs correspond in their origin to anterior or motor nerves, the superior (those nearest to the vagus) arise from the nucleus already described, and are therefore to be considered sensitive twigs, and this is shown not only by their origin from the analogue of the substantia gelatinosa, and their close relation to that structure, but also by the intimate union of the elementary fibres with those coming from the hypoglossal nucleus. According to this view the twigs given off by the spinal accessory to the hypoglossus and vagus nerves are sensitive, and derived from this portion of the medulla, while the principal or motor branch is made up of the inferior and middle filaments. Dr. Stilling says, that he has excited reflex motions in the tongue of an animal by irritating the superior twigs, but does not seem to place much reliance on his experiment.

*The hypoglossus.* Dr. Stilling has confirmed by vivisections the views he adopted à priori, respecting the functions of this nerve. On irritating the hypoglossal nucleus of cats and whelps with a couching-needle, the most distinct motions of the tongue were excited, and of its right and left side, accordingly as the left or right nucleus was irritated.

The skull of the animal experimented upon was opened, the cerebellum removed, and the walls of the fourth ventricle exposed. Dr. Stilling denies that irritation of the hypoglossi themselves will excite motions in the tongue. As the connexion between the hypoglossus, glosso-pharyngeus, and vagus is so intimate, he tried whether irritation of the latter would excite reflex action in the lingual muscles, but without effect.

*Functions of the vagus.* From Dr. Stilling's previous remarks on the anatomical origin of this nerve, we have inferred that in his opinion it belonged to the class of sensitive nerves. But he subsequently states it to be of a mixed character. This mixed function he attributes to the "lesser spinal bodies" which first show themselves in the nucleus of the vagus, and are analogous to the greater in the anterior columns. He supposes that the former may be subservient to involuntary muscular action, as the latter is to voluntary. Irritation of the vagus nucleus did not induce those movements of the viscera which irritation of the roots of the nerve excites.

The functions of the glosso-pharyngeus, like those of the vagus, are of a mixed character, (for the reasons stated respecting the preceding,) being both motor and sensitive. With regard to the general functions of these nerves, Dr. Stilling observes, that all impressions which traverse the substantia gelatinosa, from the spinal cord upwards to the medulla oblongata, excite the superior roots of the accessorii, vagi, and glosso-pharyngei, and through these the roots of the hypoglossi. We can thus easily understand how disagreeable and painful impressions excite sudden outcries, changes in the respiration, the various movements of the tongue, trunk, &c. The brain may also excite the medulla oblongata in this way from above, as well as the spinal cord from below. Dr. Stilling also remarks that the condition of the medulla oblongata will have an influence on both the brain and spinal cord, and thus we can understand why diseased states of the viscera may influence the central organs by acting on the nerves in connexion with the former. We need not enumerate the functional diseases of the brain and spinal cord that may be thus produced: tetanus, trismus, delirium tremens, hallucinations, &c. are some of those enumerated by our author. We should infer, from the preceding, that the pathological changes in hydrophobia will be found in the structures forming the floor of the fourth ventricle.

Seven plates exhibit the anatomical details (magnified fifteen times) above described, and correct the errors of the plate given in the first number of the work. It appears from our author's observations, that the latter are not worthy of credit; indeed, that is sufficiently proved by the plates themselves, for they have rather the characters of theoretic diagrams than of true representations of nature, resembling in this respect the plates of our earlier anatomical works.

The "spinal bodies" so frequently alluded to in the present number, nowhere appear in the preceding plates nor scarcely a trace of the distended capillary vessels for which they were mistaken. Dr. Stilling takes great pains to convince his readers that the present plates are done with the greatest care and accuracy; without actually impugning the latter, we certainly must suspend our judgment, simply, in fact, because they appear so very complete.

The method Dr. Stilling followed was this. First, the medulla oblongata and spinalis was placed in weak alcohol, (the membranes being removed ?) for twenty-four hours. This was then poured off, and stronger added, and then in two or three days poured off again and the strongest alcohol substituted. Of course, the mouth of the jar is closed with a bladder. In four to eight days it is sufficiently hard to be sliced for examination. This is done by a sharp and highly-polished razor; the blade must be wet with alcohol each time it is used, and the slice must be so thin that the polish of the razor can be seen through it. Dr. Stilling gives directions for obtaining this excessive tenuity, which nothing, we are certain, but frequent practice can arrive at. After a slice sufficiently thin is obtained, it is removed to a glass-plate, and wet with alcohol, &c. No compression is used, so that the structure is seen in its natural state. Dr. Stilling very handsomely offers to send slices to those physiological inquirers who cannot "cut thin" themselves.

In the next number we are promised a description of the organization of the pons varolii and cerebellum, and of the origin of the nerves in connexion with the former. Undoubtedly, the mode of examination adopted by Dr. Stilling, is a great improvement in physiological research, and we await the result of his future inquiries into the structure of the brain with some interest.

II. DR. HANNOVER'S RESEARCHES. A prize having been offered by the Royal Society of Copenhagen, for the best essay upon the "advantages and results which have accrued to physiological science from the recent microscopical investigation of the nervous system," Dr. Hannover became a candidate for the honour, and the work before us is the result of his labours. The observations detailed by him were made exclusively upon aged or adult animals, and were restricted to those which could be easily procured, in order that his investigations might be repeated. In examining the brain and spinal marrow, his attention was chiefly directed to the cells, which exhibit much more variety than the fibres. The drawings were all made from the microscope by the camera lucida, and perforated mirror fixed to that instrument in an ingenious way, and they are all of about 340 diameters.

*Microscopic anatomy of the nervous system of vertebrata.* Dr. Hannover states that the brain and spinal cord consists of two principal elements,—cells, and fibres,—the cells being vesicles consisting of a membrane enveloping fluid contents, with one or more nuclei, and the enveloping membrane, a very fine-grained substance. The cells are generally round, often oval, sometimes triangular, or one end is prolonged to a point, while the other is rounded. Their circumference varies from that of a human blood-corpuscle to that of six or twelve blood-corpuscles of the frog. Their form is much modified by pressure one against the other. The fluid contents of the cells are seen with difficulty, but the nucleus can be observed to change its place, when the object beneath the microscope is slightly shaken. The cells certainly contain a fluid, and not air. The nuclei of the cells are of a darker colour, and are defined by a sharp dark outline. Their diameter varies from that of a human blood-corpuscle to that of a blood-corpuscle of a frog, and in very large cells is even larger. There is seldom more than one nucleus in each cell.

More nuclei than cells are often met with, apparently under the microscope, for the cells are sometimes entirely filled with the nuclei. The nucleus itself is a vesicle filled with fluid. Dr. Hannover asks whether these are to be considered as complete cells of small size, or whether the nuclei are vesicles surrounded by a cell-membrane, so that one vesicle is contained within the other. Dr. Hannover inclines to the former idea.

Within the nuclei are found one or more bodies, (kjerne legern, corpora nucleorum.) Their size sometimes equals that of a human blood-corpuscle, but they are more generally mere points. The largest that he has met with were in the spinal cord of sucking animals, and from their occasional double appearance, he supposes them to be likewise vesicles. The cells of the brain are to be found wherever the cerebral substance is not entirely white, but in the purely white substance there is not a single cell.

*Fibres of the brain.* (Hjerne traadene.) They are cylindrical, and consist of a cylindrical axis, a pith or marrow, and a surrounding sheath, and are best seen in the floor of the fourth ventricle. They appear to be enveloped in a double sheath, the inner sheath lying next to the cylindrical axis. This latter often extends itself, like a fine thread, beyond the broken portion of the fibre. The most minute fibres of the brain are probably composed in like manner ; and this may be seen when the sheath of any of them becomes *rumpled* or varicose, when a small central line or cylindrical axis can be perceived, which is not affected in a similar way. The white matter of the brain is formed exclusively of fibres, but they also occur in small numbers in the gray substance, where they are much softer and more tender.

The fibres of the brain lie generally parallel, *without* forming anastomoses; and they also frequently lie in bundles, as in the corpus striatum and optic thalami, &c., as may be seen with the naked eye.

*The origin of the cerebral fibres, and their continuation into the nerves of the periphery of the body.* The fibres of the brain spring from the cells—from the cell itself, in fact, and not from its nucleus, though the latter appears to be the case when the nucleus occupies the entire cell. No more than two fibres spring from one cell, but *one* is quite as frequent. Dr. Hannover also thinks that the connexion between the fibres and the cells may cease when the former are completely formed. A torn and overhanging portion of the varicose sheath of the fibres may be mistaken for an attached cell. Nor must we mistake the tail-like prolongations of some cells for fibres.

The fibres run in the brain from the superior portion to the bases, and have consequently a perpendicular direction ; but in the layer of white matter immediately below the gray, they run parallel to the surface. Many fibres pass off to the roots of the nerves that spring from the brain; others descend into the spinal marrow, and, bending from their downward course at an obtuse angle, enter the roots of the nerves, and become nervous fibrils without interruption. The transition from the one to the other takes place rather suddenly, and Dr. Hannover states that he can discover no *real* difference of structure between the two, though the cerebral fibre differs in thickness, and is more tender, and more liable to form varicosities.

Certain fibres cross the spinal cord from one side to the other, besides those which descend perpendicularly from the brain. Dr. Hannover imagines these to end in the roots of the nerves, but acknowledges that he has had the greatest difficulty in investigating the microscopic anatomy of the spinal cord. It is certain, however, he says, that no real *crossing* of the fibres takes place in the cord.

*Arrangement of the fibres of the brain.* Dr. Hannover confirms by the microscope the observations of Remak and Baillanger, that the superficial gray matter of the brain is composed of several layers. We pass over the details exhibiting the microscopic anatomy of the brain, and spinal marrow of the perch, common frog, triton cristatus, and domestic fowl, to some details respecting that of mammalia. In the brain of large mammals and of the human species, the gray substance is seen to consist of six layers, which Dr. Hannover has figured. The fibres run horizontally on the convolutions in the external or first layer, which is exceedingly thin. The whiteness of some of these layers depends upon the deficiency of cerebral cells (ganglion-globules;) which latter present great varieties in size and form, as they occur in different parts of the brain. These varieties are described by Dr. Hannover. It appears that, with respect to the ganglion-globules of the pituitary body in man, the prolongations of the larger cells are very remarkable. Dr. Hannover has found no fibres in any of the glands.

In the lower animals (frogs) Dr. Hannover has seen ciliary movements in those parts that lie contiguous to the cavities of the brain, and he has often also observed an undulatory motion, where the cilia (flimmerhaar) could be seen. He does not believe in the ciliary motions reported to be observed in the nerves.

In discussing the formation of the fibres of the cerebro-spinal nerves, Dr. Hannover states, that the component parts of the fibres, as observed in the brain, are still more marked in the nerves. The fibres of a nerve, immediately after being taken from the body, only appear thin and transparent, bounded by two darker lines; but as the pith before alluded to coagulates, then the axis or central canal becomes visible, and the whole appears more opaque. The central canal Dr. Hannover considers to be a hollow cylinder. The inserted figure in p. 39 is to prove this; but Dr. Hannover saw it only once, and therefore wisely declines to assert this as a fact.

*Microscopic anatomy of the ganglionic system.* The ganglionic nerves consist of three nervous elements, viz. cerebro-spinal nervous fibres, ganglion-cell or globules, and vegetative nervous fibres. The proportions of these vary greatly; the cerebro-spinal nervous fibres existing only in very small proportion in the ganglia, and are entirely wanting in the ramifications that spring from thence, while the presence of cells (ganglion-globules) first announces the existence of a ganglion. The number of the cerebro-spinal nervous fibres is greater in the cerebro-spinal than in the sympathetic ganglia.

The ganglion-globules are observed in ganglia of every size. Dr. Hannover considers them as distinct from those of the brain. They consist of an enveloping membrane, its contents, and one or more nuclei, and nucleus-corpuscles. The membrane of the cell or globule appears to consist of small (perhaps hexagonal) plates, which give a good deal more consistence to the globule than is possessed by those of the brain.

Their form is generally round or oval, seldom with any caudal prolongation. The contents of the globule are probably the same as in those of the brain: the nuclei are darker coloured, and often more numerous, especially in fish. The "vegetative nervous fibres" are found in the ganglia and in the nerves proceeding from them, and are distinct from the cellular tissue of the ganglia. They are very tough and pale, and do not exhibit any lines internally. Dr. Hannover has never observed an axis-cylinder (the term is expressive) in them; but he thinks that he has perhaps detected a cavity in those of fishes. A number of nuclei are visible upon these fibres: they are round, oval, or oblong, and their outline dark and sharp. They are often connected with very fine fibrils, and appear as if placed within a very fine sheath. The vegetative nervous fibres are spun round the ganglia, so that we see the same fibre several times. They spring from the cells (ganglion-globules) as a caudal prolongation, and many arise from one cell.

To determine the distribution and termination of the nerves in the muscles, Dr. Hannover made his observations chiefly upon the muscles of the eyes of frogs, and of the water-newt, as, from their small size, the whole muscle could be brought at once under the field of the microscope. He is of the commonly-received opinion, that the nervous fibres form loops *upon* the muscular fibres, and that they terminate in this manner. In the recti oculorum of frogs he fancies that he has discovered a curious numerical proportion between the nervous loops, and the number of muscular fibres, the latter being double the number of the loops, or *equal* to that of the *arms* of the said loops. The relative numbers are given at p. 40.

In prosecuting his inquiries as to the distribution and termination of the nerves of the skin, Dr. Hannover examined the membrana nictitans of newly-hatched birds (pigeons, &c.) He found that the nerves supplying the skin terminate either in large loops, or else end suddenly, sometimes in a point, and sometimes they remain of a similar thickness as the rest of the fibre, or they are thinner, smaller, and are pointed or rounded off. The ultimate distribution is often in one of the glands of the skin, in which case they exhibit the same structure as before described; but the internal pith and the size of the central canal? (axis-cylinder) appear to diminish gradually. Dr. Hannover confirms, by the microscope, the opinion, that both the cerebral and spinal nerves send branches from one side of the body to the other.

The nerves of smell and sight consist of simple cerebral fibres; those of hearing and of taste consist of cerebro-spinal nervous fibres, of the same character as those found at the roots of other nerves. Dr. Hannover has never been able to trace the terminations of the nerves, in the tongue, or in the nasal cavity; the density of the mucous membrane not permitting him to follow the fibres into the papillæ of the tongue. The retina and its cerebral matter were examined in all the vertebrata, excepting man; for the human eye can rarely be obtained fresh enough for microscopic examination, and no portion of the body changes so rapidly after death.

The peculiar structure of the retina is best seen in fish (especially in the pike, Esox lucius) and in these cold-blooded vertebrata, the eye will remain for twenty-four hours fit for use. Dr. Hannover separates the retina into two portions, the true retina, and its coating of cerebral matter. After scraping off the pigment *from without*, the true retina is seen to consist of two distinct elements, viz. 1, prisms sharp at one end, and blunt at that turned inwards towards the central part of the eye. These Dr. Hannover terms "*Staavene*," staves or rods, prismata peracuta, &c. When single, they appear round, when in numbers and pressed together, they are generally hexangular. The points break off after a short time, and the form is altered. The other portion or element of the retina, as seen from the outside, are the double cones (coni gemini). The innermost portion of these cones, viewed with reference to their position, is the single part or root; this appears more polished than that portion which is directed outwards, and which consists of two cones inserted into the hexangular cells of the pigment. The relative positions of these two elements upon the retina are as follows: The prisms surround the coni gemini as with a stockade. This appearance may be exhibited by scraping off both these bodies and exposing their roots, which then present a beautiful and regular mosaic work. The retina of birds, when placed under the microscope, exhibit beautiful colours, caused apparently by globules of an oleaginous matter, which strongly reflect the light. Dr. Hannover has never found in the vertebrata that the optic nerve terminates near the ciliary processes by plexuses or loops; he has always observed them to disappear gradually in the immediate neighbourhood of those processes. He also denies altogether the existence of the membrana jacobi in the animals which he has examined.

The investigations of our author into the distribution of the portio mollis are next detailed; and then the results of his researches into the microscopic anatomy of the embryo of various animals, and of several invertebrata, as the astacus fluviatilis, helix nemoralis, libellula grandis, hirudo medicinalis, &c. Seven plates, containing ninety figures (some of which are coloured), illustrate the details in the text. Altogether, the book, if not containing much which is absolutely new, displays great industry on the part of its author, and is valuable as corroborating many doubtful points in neurology. We can only complain that it is written in Danish, and we do this as much for the sake of the author as for our own. Very few Europeans indeed, out of Denmark, read Danish.

III. DR. MEYER'S RESEARCHES. The researches of Dr. G. H. Meyer into the physiology of the fibrils of nerves, are literary and not experimental. His volume is one of the works which are professedly written for the express purpose of arranging the new facts connected with the subject communicated to the world from time to time, and placing them in new and varied relations to each other. Dr. Meyer has a theory of his own, for of course a connecting link of theory is as necessary to literary and meditative researches, as it is to experimental.

*General physiology of the nervous system.* Dr. Meyer's object, in the present work, has been to consider the fibril in its elementary and immediate function rather than in the complex phenomena it displays when collected into masses. He considers the latter, however, by arranging the nervous fibrils into two groups, the peripheral and the central. The peripheral is divided into motor and sensitive, animal and sympathetic fibrils. Dr. Meyer makes the nervous fibrils of the vascular system a subdivision of the motor, being of opinion, however, that they belong both to the animal and sympathetic system.

Neither our author nor our readers will expect us, we hope, to give a detailed analysis of a work comprising a review of the whole of modern neurology, and compressed into 316 small, closely-printed, octavo pages. It will be better, perhaps, to refer generally to the works before us, and inquire how far the facts detailed elucidate certain obscure points in this branch of natural science. It is generally taught that there is a gradation in the development of the nervous system of animals from the lowest radiata to the mammalia, and from the lowest mammal to man. Physiologists have traced the points of connexion of each animal with the other with an enthusiasm not dissimilar to that which inspires the antiquary. The striking analogies between portions of the osseous, vascular, muscular, and nervous systems in each and every class have been pointed to as proving a unity of plan throughout the whole. Now, if this leading doctrine of transcendental physiology be true, (and we think it is,) the nervous system of animals must be studied as one whole, and the structure and function of distinct classes and tribes be elucidated by the knowledge derived from this study. In this respect, the works before us present little information. If the nervous system of the polype, or of the radiata, be analogous to the sympathetic system of mammals, our knowledge of the former is in no respect made to elucidate the latter. This doctrine of unity of plan has another important application. A priori, a knowledge of the structure and function of the sympathetic and its ganglia ought to throw light on the texture and functions of the spinal cord; and a knowledge of the texture and function of the spinal cord should elucidate cerebral anatomy and physiology. Now, we certainly do not find this application of the doctrine alluded to systematically carried out in the works before us. Isolated facts are mentioned, however, proving that it has not been quite neglected. Bidder and Volkmann could detect no difference between the fibrils of the cerebro-spinal nerves and sympathetic nerves of the embryo calf, even with the best microscope, and we have seen that Purkinje and Rosenthal inferred from their researches that the cerebro-spinal nerves of the fœtus were not different from the permanent sympathetic nerves of the adult, and that the latter should be considered as a lower grade of development of the former. Again, both Van Deen and Stilling insist upon the theory that the spinal cord is a chain of ganglia, as we know its analogue in the articulata to be, basing this theory on the general fact established by their experiments, that frustra of the spinal cord have the functional activity, proper to the whole cord, complete within themselves. Now this we know to be true also of the vermes and articulata. Sections of the spinal cord of the frog are capable of originating combined movements, so also sections of the spinal ganglia of articulata. Again, the connexion of the motor power of the heart and abdominal viscera with the spinal cord, is undoubtedly elucidated by the experiments of Budge; they confirm, by cruel vivisections, what we knew already by the effects resulting from the influence of the passions, &c. The automatic motions observed in animals of the lowest grade are reproduced in the organs of mammals connected with the sympathetic system. These motions, essentially the same, although phenomenally different, are reproduced again in the automatic or reflex movements of the spinal cord, consciousness being superadded or not, just as the case may be; but if superadded, complicating the phenomena, and connecting them with the physiology of the encephalon.

And then, with regard to the physiology of the brain—what inference can we draw, or rather can we avoid drawing from the doctrine alluded to? Is it not that the analogue of cerebral structure and function is in spinal structure and function? And if so, what is the analogue of that so-called reflex action in the spinal cord which mimics the motion excited by the will and guided by the understanding? The field of conjecture and experiment which this view opens to both the physiologist and pathologists, is boundless. The pathology of insanity, and other results of disturbed cerebral action, of animal magnetism, and of other changes not rigidly to be termed morbid, may possibly be both elucidated and explained to an unhoped-for extent, by classing their phenomena with the reflex phenomena of the spinal cord. Nor would the good results end there, for as the brain is the organ of mind, mental philosophy would necessarily participate in the general progress. The instinctive actions of the lowest animals are reflex in character; are those of mammals less so? If not, then this is the route by which we should seek knowledge respecting the physiology of the passions.

Meyer advocates some of these views directly or indirectly, although his ideas are given too much in the Kantian jargon, apparently in deference to immaterialism, to be intelligible. Meyer argues also that there is no real difference between the motor and sensitive nerves, and with some plausibility. We will not venture to state the analogies which support his notion. The resemblance between the ganglion-cells of Hannover, and the "spinal bodies" of Stilling, is too striking to be passed over unnoticed. There are one or two other points of detail, to which we would shortly refer. It will be seen from Stilling's researches, that the elementary fibrils of the nerves interlace with each other in the spinal cord. Now, what takes place within the cord, takes place more visibly without; indeed, we think that the various plexuses of the spinal nerves strongly prove the accuracy of this part of Dr. Stilling's researches. It is precisely analogous to what is seen in the venous and lymphatic systems. There we find small branches uniting with larger, and those into still larger, until they constitute a main trunk. The minuteness of the component parts is indeed merely relative; what we see with the naked eye to exist is only that on a larger size which we observe under the microscope.

But how can we understand that continuity of structure between the central and peripheral extremities of motor and sensitive nerves generally supposed by physiologists to exist? If there be the intimate intermixture stated, first in the ultimate distribution of the nervous fibrils, next in the plexuses usually described, then again within the spinal cord, and in a mode analogous to that observed in the ultimate distribution, and again very probably within ganglia after ganglia in the encephalon, a structure necessary indeed to the production of those multiform phenomena which a single impression on a sensitive nerve will produce, how can be understood the isolated and perfect continuity of the nervous fibrils in their whole course? To increase the difficulty still more, we find that lower animals, as vermes, arachnida, and certain species of reptiles, have whole limbs reproduced, and in man himself, new portions of resected nerve are formed with little or no disturbance of the motor or sensitive powers. Without venturing upon a decided opinion, we may here at least observe, that this doctrine of isolated conduction and continuity, so commonly asserted as undeniably true, requires further investigation, and such modification as will harmonise it with the results of more recent anatomical research.

